# Effectiveness of a Single Education and Counseling Intervention in Reducing Anxiety in Women Undergoing Hysterosalpingography: A Randomized Controlled Trial

**DOI:** 10.1155/2014/598293

**Published:** 2014-01-16

**Authors:** Alfredo La Fianza, Caterina Dellafiore, Daniele Travaini, Davide Broglia, Francesca Gambini, Luigia Scudeller, Carmine Tinelli, Edgardo Caverzasi, Natascia Brondino

**Affiliations:** ^1^Department of Radiology, IRCCS Policlinico San Matteo, University of Pavia, Viale Golgi 19, 27100 Pavia, Italy; ^2^Department of Brain and Behavioral Sciences, University of Pavia, Via Bassi 21, 27100 Pavia, Italy; ^3^Scientific Direction, IRCCS Policlinico San Matteo, 27100 Pavia, Italy

## Abstract

Hysterosalpingography (HSG) is generally considered a stressful and painful procedure; we aimed to evaluate whether a single education and counseling intervention could reduce women's distress and pain after undergoing HSG for infertility. Patients were randomized into control group (*n* = 108) and intervention group (*n* = 109). All patients filled the following questionnaires before and after HSG: Zung self-rating anxiety scale (Z-SAS), Zung self-rating depression scale (Z-SDS), and an ad hoc questionnaire designed to evaluate HSG procedure knowledge. Pain was scored using a visual analog scale. The intervention consisted in a 45-minute individualised session 48 h before HSG. We observed a reduction of anxiety and depression scores in the intervention arm compared to the control group. After controlling for potential confounding variables, intervention was an independent predictor of the difference of Z-SAS score before and after HSG. This is the first randomised controlled trial to assess the potential effectiveness of a single education and counseling intervention to lower anxiety in a diagnostic setting.

## 1. Introduction

In the industrialized countries, approximately 6.6–26.4% [1] of couples of reproductive age are confronted with infertility, which is clinically defined as the failure to achieve a pregnancy after 12 months or more of regular unprotected sexual intercourse [2, 3]. The potential causes of female infertility are numerous and may involve the fallopian tubes, peritoneum, endometrium, uterus, cervix, and ovaries. Fallopian tube abnormalities account for 30%–40% of cases of female infertility [4]. Hysterosalpingography (HSG) is part of the diagnostic routine of the infertility workup and, despite the development of other diagnostic tools such as MR imaging, is considered the gold standard in the assessment of the patency of the fallopian tubes [5, 6]. HSG is an invasive procedure and is generally regarded as uncomfortable and painful: 85% of women reported pain during HSG, with half complaining of moderate to severe pain [7, 8]. It had been observed that level of anxiety and distress experienced by patients correlates with the level of invasiveness of the procedure: in particular, state anxiety in women awaiting HSG was significantly higher compared to women awaiting mammography or abdominal ultrasonography [9]. Women undergoing HSG are found to experience considerable stress before and during this examination [10]; additionally, infertility in itself is known to cause prolonged stress and distress for the couples involved [11].

Some research supports the benefits of psychological interventions in reducing psychological symptoms in both infertile women and patients undergoing painful procedure. It has been shown that psychotherapy and counseling interventions led to significant decreases in anxiety and depression and increases in the chance of pregnancy in infertile patients [12–15], even if levels of emotional distress before assisted reproductive technology seem to not influence the chance of becoming pregnant [16]. Additionally, nonpharmacologic practices, such as guided imagery, music therapy, hypnosis, and distraction, can effectively improve patient experiences during painful medical procedures, such as endoscopy [17, 18].

So far, to the best of our knowledge, no study has examined the effect of a single education and counseling intervention on women undergoing HSG. The aim of this randomized study is to evaluate whether a single education and counseling intervention, when compared with routine care, could decrease women's distress and the effects of distress on the ease of performing HSG for infertility diagnosis.

## 2. Materials and Methods

### 2.1. Subjects

Between November 2008 and September 2010, 217 women requiring HSG for infertility workup agreed to participate in the study. The diagnosis of infertility was done by a senior gynaecologist. Patient exclusion criteria were as follows: presence of psychiatric disorders, use of psychotropic medications, and poor understanding of Italian. Written informed consent was obtained from all participants. The study was approved by the local ethics committee and was performed in accordance with the Declaration of Helsinki. Participant baseline characteristics, including demographic information (i.e., age and gender), clinical data, and mental health history, were collected. Following random allocation into the control and intervention group, women in both groups were assessed by means of the Zung self-rating anxiety scale (Z-SAS) and Zung self-rating depression scale (Z-SDS) to establish baseline anxiety and depression levels. Both groups were reassessed immediately after HSG using the same questionnaires. Additionally, each women completed a questionnaire evaluating pain perception.

### 2.2. Randomization

After providing informed consent, patients were randomized into control group (*n* = 108) and intervention group (*n* = 109, receiving counseling), using computer-generated random numbers. Numbers were placed in opaque envelopes, and group allocation was managed by an independent administrator. Both the radiologist and the technician who performed HSG were blinded to the patient's allocation.

### 2.3. Hysterosalpingography

Hysterosalpingography was performed by a senior radiologist and a technician. A balloon catheter is threaded into the cervix or uterus. Water soluble iodinate nonionic contrast medium was injected through the catheter and radiographic images of the uterus and fallopian tubes were obtained. Oral premedication with anti-inflammatory drugs, analgesics, or local anaesthetics was not administered.

### 2.4. Intervention

The education and counseling intervention was performed by three qualified psychotherapists from the university staff.

Women assigned to the intervention arm underwent a 45-minute individualised session 48 h before HSG. Therapists were specifically trained to provide a health education component consisting of information about HSG procedure and its potential painfulness. The therapist provided a method for stress management focusing on improvement of family support, effective problem solving, and personal coping. All individual sessions addressed the aforementioned items. Additionally, where relevant, specific issues associated with infertility were addressed with exploration of patients' beliefs about this condition and their expectation about HSG results. Psychological support was available from the support climate provided by discussion between patient and therapist. No intervention was carried on in the control group.

### 2.5. Primary Outcome

The primary outcome measure was the change of the Z-SAS scores before and after the intervention. Z-SAS is a self-administered scale, consisting of 20 items [19]. Each item is scored on a four-point scale (from 1 to 4), according to severity. A higher score denotes more serious anxiety disorder. Scores above 36 are considered to identify “clinically significant” anxiety [20].

### 2.6. Secondary Outcomes

The Z-SDS is generally considered a reliable instrument for measuring depressive symptoms in primary care [21]. It is a self-reported 20-item questionnaire. Items responses are rated from 1 to 4, with higher scores corresponding to more frequent symptoms. The overall score represents the severity of depressive symptoms. A cut-off score of 40 indicates “clinically significant depression” [22].

Patients filled an ad hoc questionnaire designed to evaluate HSG procedure knowledge.

The pain was scored using a visual analog scale (VAS) [23]. The patients were asked to indicate a point along a 10 cm continuous line from 0 to 10 (no pain to excruciating pain). The distance, measured in cm (to the nearest 0.1 cm) of the marked point from the 0 edge provided the VAS score.

### 2.7. Statistical Analysis

The planned sample size (100 patients per group) was calculated to detect a difference of 10 points in the anxiety scale in the treated group, in the hypothesis of mean 45 points in the untreated group, and a common standard deviation of around 23 points, with power 80% and alpha error 5% by a Student's *t*-test for independent samples.

Data were checked for normal distribution using the Kolmogorov-Smirnov statistics. Normally distributed data and skewed variables are presented as means ± SD or medians and interquartile ranges, as appropriate. Prior to the main analyses of the effect of the intervention, the two groups were compared with respect to their sociodemographic characteristics, infertility characteristics, reproductive history, and baseline psychological profiles to determine if the randomization was successful. Student's *t*-test (rank sum test or Mann-Whitney test for skewed distributions) was used in the two groups to compare quantitative variables (ANOVA or Kruskal-Wallis for >2 groups, resp.). Categorical variables were compared using chi-square test or McNemar's test. Pearson's or Spearman's correlation coefficient was used to determine relationship between variables. Mixed design ANOVA was used to compare changes of anxiety and depression scores in the intervention and the control group. Linear multiple regression analyses were used with mean differences in anxiety and depression scores as the dependent variables and baseline levels of anxiety and depression, intervention, age, months of infertility, marital status, family history of psychiatric disease, and reproductive history as the independent variables.

All statistical analyses were carried out using SPSS version 16.0 (SPSS Inc., Chicago, IL, USA) and GraphPad Prism version 4.0 (GraphPad Software Inc., San Diego, CA, USA). A two-tailed *P* value < 0.05 was considered statistically significant.

## 3. Results

### 3.1. Sample Characteristics

A total of 217 women chose to participate. Of the 109 subjects in the intervention arm, one eventually did not undergo HSG and was excluded from the analysis, leaving 108 women in the intervention arm and 108 in the control group. The sociodemographic and clinical characteristics of the two study groups are shown in Table 1. For the majority of the sample, women were of Italian origin and married. The 32.2% (*N* = 67) had a tertiary education level.

Univariate analyses (*t*-tests for independent samples) showed statistically significant differences between intervention and control group regarding the Z-SAS baseline score (31.21 ± 5.66 versus 28.89 ± 6.54, *t*(215) = 2.73, *P* < 0.05) and the Z-SDS baseline score (31.72 ± 7.35 versus 29.31 ± 7.65, *t*(215) = 2.32, *P* < 0.05). However, the intervention and the control arm were not significantly different with regard to the number of clinical anxiety cases (17.5% versus 14.4%, chi-square = 0.36, *P* = 0.50) or clinical depression cases (13.6% versus 9.4%, chi-square = 0.89, *P* = 0.39). No significant difference was observed between the two groups regarding knowledge about HSG. Baseline anxiety and depressive symptoms scores were inversely correlated with years of education (*r* = −0.22, *P* = 0.01 and *r* = −0.17, *P* = 0.01, resp.) and age (*r* = −0.16, *P* = 0.01 and *r* = −0.14, *P* = 0.03, resp.). Pain perception was inversely correlated with age (*r* = −0.16, *P* = 0.02).

### 3.2. Primary Outcome/Changes in Anxiety Scores

**Figure 1:**
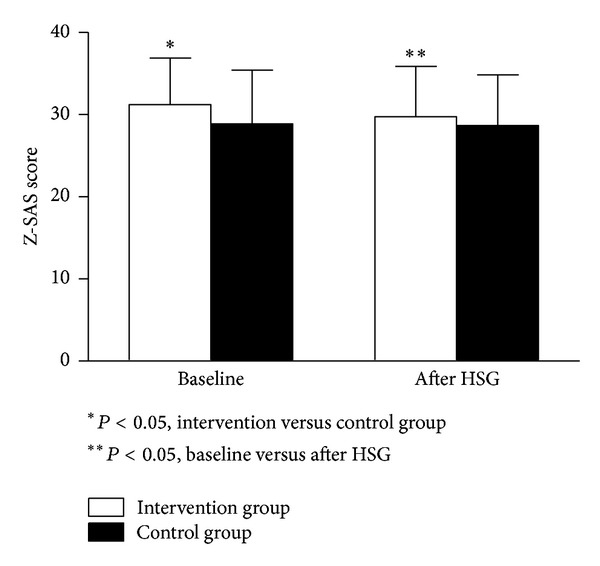
Changes in Zung self-rating anxiety scale (Z-SAS) before and after hysterosalpingography (HSG) in the intervention and the control groups. Scores above 36 identify clinically significant anxiety.

Changes in Z-SAS before and after HSG are depicted in Figure 1.

Mixed between-within-subjects ANOVAs indicated that there was an effect of time on Z-SAS scores from before to after HSG (*F*(1,203) = 7.102, *P* = 0.01), as well as a significant main interaction (group × time) (*F*(1,203) = 5.044, *P* = 0.01). The results indicated a reduction of anxiety scores in the intervention arm compared to the control group.

### 3.3. Secondary Outcomes/Changes in Depression Scores

**Figure 2:**
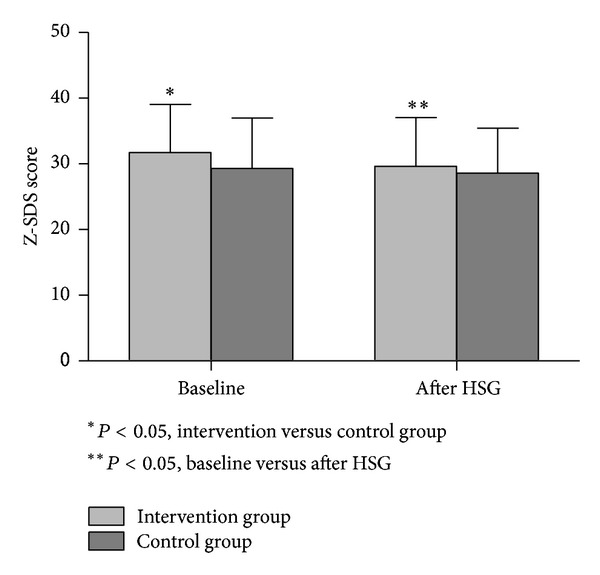
Changes in Zung self-rating depression scale (Z-SDS) before and after hysterosalpingography (HSG) in the intervention and the control groups. Scores above 40 identify clinically significant depression.

Changes in Z-SDS before and after HSG are depicted in Figure 2.

Mixed between-within-subjects ANOVAs indicated that there was an effect of time on Z-SDS score (*F*(1,201) = 18.68, *P* < 0.01), as well as a statistically significant main interaction (group × time) (*F*(1,201) = 3.91, *P* = 0.04). The results indicated a reduction of depressive symptoms scores in the intervention arm compared to the control group.

According to the Zung definition of clinical anxiety and depression, a reduction of number of depression cases in the intervention group (McNemar's test, *P* = 0.04) was observed.

### 3.4. Secondary Outcomes/Pain during HSG

No significant differences were found in pain perception between the intervention and the control group (5.22 ± 2.67 versus 5.00 ± 2.57, *t*(215) = 0.57, *P* = 0.50).

### 3.5. Multivariate Analysis

Multivariate regression analyses (Table 2) showed that, after controlling for potential confounding variables, the education and counseling intervention was an independent predictor of the difference of Z-SAS score before and after HSG; additionally, baseline anxiety and marital status were independent predictors of the difference of Z-SAS. The only independent predictor of the difference of Z-SDS score before and after HSG was the level of baseline depressive symptoms.

## 4. Discussion

This randomized controlled trial has sought to test the effectiveness of education and counseling as a brief intervention in reducing distress symptoms among women undergoing HSG. This study found a significant difference in anxiety and depressive symptoms scores between the intervention and the control group. The current findings are consistent with those of a previous study in which 120 patients undergoing carotid endarterectomy (an invasive procedure) were randomized to receive structured information and counseling the day before surgery or standard preintervention counseling [24]. Adequate information regarding the procedure and counseling effectively reduced the level of anxiety. However, there are some differences between the studies. Baseline anxiety scores in patients before endarterectomy were higher than in women undergoing HSG. This datum could be ascribed to the different level of invasiveness associated with the procedure. Moreover, endarterectomy itself presents a higher risk of potential severe complications compared with HSG, which could have contributed to the baseline higher anxiety levels of patients in the study of Yang et al. We also noted a negative association between educational level and anxiety and depression symptoms (patients with higher education displayed lower levels of anxiety and depression symptoms), which is in line with previous researches [25]. Therefore, the observed lower level of anxiety could be explained by different demographic characteristics of the two populations. Additionally, the control group in our trial did not receive any type of education and counseling, thereby allowing for more direct comparison of treatment effects. Moreover, our results are in line with the studies of Balci and coworkers [26] and Walsh et al. [27] which showed that patient education was effective in reducing anxiety and pain in women undergoing amniocentesis or colposcopy, respectively. Counseling was able to reduce distress scores even in women undergoing mammography [28]. In contrast, some studies [29–31] did not reported a significant effect of patient education on anxiety in women before an invasive procedure. However, it could be argued that, in all the aforementioned trials, the psychological and educational intervention consisted only in information provided by videos or leaflets. Only one study [29] offered a structured discussion following the video. It could be suggested that even the most well-produced videos will not be entirely effective if patients are prevented from discussing or asking questions. In fact, some studies have underlined the important role of the counsellor/nurse in lowering anxiety in women before assisted reproduction technique [32–34]. However, few studies have directly compared face-to-face counseling to more impersonal (videos, leaflets, and websites) forms [35, 36]. Our data might strengthen the importance of the contribution of the counsellor in clinical settings [37].

Interestingly, we did not find a reduction in pain scores in the intervention group: in fact, both groups reported higher level of pain compared to women undergoing colposcopy or amniocentesis [26, 27]. It could be hypothesized that HSG is eventually a painful procedure and that education and counseling alone could be not sufficient in controlling pain levels.

Several limitations of the findings should caution against overinterpretation. Firstly, the randomization did not result in equity between the groups for anxiety and depression scores at baseline. Though, even if we controlled for these variables by means of multiple regression analysis, it is possible that our findings could have been biased. Secondly, even if statistical power was adequate to detect anxiety level and depressive symptoms changes, our sample did not allow us to identify predictors of treatment response. Another limitation could be ascribed to subject self-selection: patients who agree to take part in this study may be more likely to report therapeutic gains than those who choose not to participate. Additionally, we offered a single education and counseling session. However, several studies have shown that brief counseling appeared as effective in reducing anxiety as longer treatments [24, 38–40]. Notwithstanding, further research with longer duration of education and counseling, together with a cost effectiveness analysis, may be needed to better elucidate the effect of information and counseling for reducing anxiety in women undergoing HSG. Additionally, our control group was a no-intervention group: it could be possible that a control group with a 45-minute session of unspecific counseling could be more useful in order to discern the effects of time and conversation per se as compared to a structured, goal-oriented intervention.

## 5. Conclusion

To our knowledge, this is the first randomised controlled trial to assess the potential effectiveness of a single education and counseling intervention to lower anxiety and depression in a diagnostic setting. Our data might cautiously suggest that patient education and counseling could exert a potential beneficial effect on anxiety and depression in women undergoing HSG, although these preliminary results clearly need replication with a larger sample. Intriguingly, future studies may test the efficacy of group education and counselling intervention: in fact, group sessions are extremely cost effective and allow participants to learn not only from the counselor/teacher but also from other patients in a similar situation, thus strengthening social and emotional connections between subjects with similar experience and difficulties.

## Figures and Tables

**Table 1:** Baseline sociodemographic and clinical characteristics of intervention (*n* = 108) and control (*n* = 108) groups.

	Intervention (*n* = 108)	Control (*n* = 108)	*P* value
Sociodemographic information			
Age (mean ± SD)	34.38 ± 4.96	34.59 ± 4.77	0.75
Marital status, married (%)	75 (71.4)	80 (76.2)	0.62
Italian origin (%)	102 (93.6)	99 (91.7)	0.60
Education (years)	14.29 ± 4.03	14.54 ± 3.85	0.65
Primary (%)	23 (21.7)	22 (21.6)	0.42
Secondary (%)	53 (50)	43 (42.2)	0.43
Tertiary (%)	30 (28.3)	37 (36.3)	0.42
Reproductive history			
Previous pregnancies (deliveries) (%)	28 (24.7)	31 (29.2)	0.79
Previous extra-uterine pregnancies (%)	2 (1.9)	4 (3.8)	0.44
Previous spontaneous abortions (%)	31 (28.8)	24 (22.6)	0.44
Prior gynaecological surgery	31 (28.4%)	33 (31.7%)	0.60
Positive family psychiatric history	25 (23.1%)	16 (14.81%)	0.10
Pain Perception	5.22 ± 2.67	5.00 ± 2.57	0.50
Clinical anxiety cases			
Baseline (%)	18 (17.5)	15 (14.4)	0.50
After HSG (%)	16 (14.8)	13 (12)	0.50
Clinical depression cases			
Baseline (%)	14 (13.6)	10 (9.4)	0.39
After HSG (%)	7 (6.4)*	5 (4.6)	0.50

HSG: hysterosalpingography.

*Significant reduction between baseline and after HSG in the same group, *P* < 0.05.

**Table 2:** Multivariate linear regression analyses for the changes in Z-SAS and Z-SDS scores before and after HSG.

Variables	Multivariate regression for differences in			Multivariate regression for differences in		
	Z-SAS score			Z-SDS score		
	*B*	SE	*β*	*B*	SE	*β*
Intervention	−1.44	0.59	−0.16*	−0.83	0.69	−0.08
Baseline anxiety/depression	−0.24	0.04	−0.39*	−0.20	0.04	−0.34*
Marital status (single versus married)	−1.79	0.70	−0.17*	0.06	0.82	0.01
Years of education	0.01	0.08	0.08	0.06	0.09	0.05
Family history of psychiatric disorders	1.12	0.74	0.10	0.07	0.85	0.01
Age	−0.04	0.06	−0.04	−0.17	0.07	−0.16

Z-SAS: Zung self-rating anxiety scale; Z-SDS: Zung self-rating depression scale; HSG: hysterosalpingography; SE: standard error; *B*: unstandardized coefficient of regression; *β*: standardized coefficient of regression.

*P < 0.05.
